# Investigating the Impact of Lived Experience Stories on Self-Harm, Mood, and Help-Seeking Intentions: Web-Based Between-Participants Experimental Study in Individuals With Recent Self-Harm

**DOI:** 10.2196/71280

**Published:** 2026-03-04

**Authors:** Jennifer Ferrar, Lizzy Winstone, Ian Penton-Voak, Lucy Biddle, Paul Moran, Lydia Grace, Becky Mars

**Affiliations:** 1 School of Psychological Science University of Bristol Bristol United Kingdom; 2 Centre for Academic Mental Health Population Health Sciences, Bristol Medical School University of Bristol Bristol United Kingdom; 3 NIHR Biomedical Research Centre University Hospitals Bristol and Weston NHS Foundation Trust Bristol, England United Kingdom; 4 Samaritans Surrey United Kingdom

**Keywords:** help-seeking behavior, internet-based intervention, mental health recovery, personal narratives, self-injurious behavior

## Abstract

**Background:**

Lived experience stories are often used on formal help sites as a support resource for individuals who self-harm. While self-harm–related internet use provides an alternative for individuals who are not yet ready or are unwilling or unable to access support offline, it has also been shown to unintentionally reinforce self-harm behavior. There are several components that might influence whether a lived experience story is perceived as helpful, unhelpful, or potentially harmful, and the evidence supporting that these encourage help-seeking in the reader is limited.

**Objective:**

This study is part of a mixed methods project that aimed to investigate how variations in help-seeking messages contained within online lived experience stories are interpreted by and psychologically impact those with a history of self-harm.

**Methods:**

Individuals with a recent history of self-harm were recruited via newsletters, social media, and websites run by the university and mental health charities to take part in an online experiment. During the experiment, participants were randomized to read stories that either mentioned (1) self-help strategies, (2) seeking help from informal and formal sources, or (3) did not mention help-seeking. Help-seeking intentions, mood, entrapment, and expectations of future self-harm were measured, and participants provided feedback on the stories.

**Results:**

There was limited evidence for an effect of story type on help-seeking intentions (*F*_2, 230_=4.2; *P*=.02; η^2^=0.25), and clearer evidence for an effect of story type on negative affect (*F*_2, 230_=4.02; *P*=.02; η^2^=0.10; adjustment for age, gender, and help-seeking history included). Participants in the “self-help” condition (n=83) reported lower negative affect after reading the stories compared to participants in the “no help” condition (n=80; mean difference=–3.97, 95% CI –7.72 to –0.22; *P*=.04) and the “informal/formal” help condition (n=75; mean difference=–3.70, 95% CI –7.55 to 0.14; *P*=.06). A key criticism of the stories was that they were unrelatable, but this sentiment was less prevalent among those in the “no help” condition. Key positives were that the stories included a realistic but hopeful outlook of recovery (less prevalent in the “informal/formal help” condition) and were supportive (less prevalent in the “no help” condition).

**Conclusions:**

While the inclusion of self-help strategies in a lived experience story reduced its impact on negative affect, the inclusion of self-help strategies or seeking help from others did not encourage help-seeking intentions. Making stories relatable, authentic, and providing multiple strategies for support might be key to encouraging help-seeking, but more research is needed.

## Introduction

Individuals experiencing self-harm or suicidal thoughts often seek support via the internet. Self-harm–related internet use can be both helpful and harmful [1,2]. Self-harm–related internet content can foster a sense of community, thereby reducing feelings of loneliness and isolation [3-6], and provide practical advice and moral support on how to manage self-harm [4,7-10]. In addition, self-harm–related internet use provides an alternative for individuals who are not yet ready, or may be unwilling, or unable to access support offline [11-14].

However, there is also a link between self-harm–related internet use and higher levels of suicidal intent [12,15,16], although the direction of effects is not clear. Due to a lack of regulation, content encouraging self-harm is easily accessible online [2,17,18]. In addition, content or environments intended to discourage self-harm could unintentionally reinforce self-harm behavior, for example, via normalization or competition [18,19]. Further, the categorization of self-harm content as either harmful or helpful is not straightforward. A recent systematic review illustrates the complex nature of engagement with self-harm content. Whether the content negatively or positively affects the individual engaging with it depends on the content, the individual, and the time and space in which it is encountered [2].

The internet provides a wide range of self-harm content, including information about self-harm behavior, self-help strategies, lived experience stories, images of self-harm, signposting to help, and dialogue [2,13,18,20,21]. They all come with limitations; for example, research suggests that information-giving can often be impersonal, disengaging, and “insufficient in moments of crisis” and that signposting can often be redundant, as many searching for help online are doing so because of barriers to engaging with signposted services [13]. The impact of self-harm images, such as scars, can be mixed, reminding some of recovery while triggering others [2]. Among those who access self-harm–related internet content, there seems to be a preference for real-time help (eg, “live chat”), self-help tools, and interaction with others, particularly via lived experience content [13,22].

Lived experience stories are often used on formal help sites. They are viewed as personal and engaging and can validate the crisis for readers, while also offering reassurance that they are not alone in their struggle and that recovery is possible [13,22,23]. However, there are several components that might influence whether a lived experience story is perceived as helpful, unhelpful, or potentially harmful. Stories appearing on forums, even those that are run by trained moderators, might be triggering for some people, for example, by including details of methods, injury, or graphic imagery. Stories appearing on websites run by charities and health care organizations usually include a personal narrative of help-seeking and recovery. However, the evidence supporting that these encourage help-seeking in the reader is limited [22,23]. Further, the careful regulation of content might be perceived as overly censored and lead some to search for unrefined content in covert and riskier spaces [2]. Even unregulated content posted by a lay individual is curated in a different respect, for example, on social media platforms where users are often presenting themselves in an idealized way [24,25].

This study is part of a mixed methods project that aimed to investigate how variations in help-seeking messages contained within online lived experience stories are interpreted by and psychologically impact those with a history of self-harm. This study used an experimental design to investigate the psychological impact of stories with different types of help-seeking messages, including those mentioning self-help strategies, informal/formal help-seeking (ie, friends, family, or professionals), and no help-seeking. We sought to assess if the type of help-seeking referenced in a lived experience story had an impact on help-seeking intentions, mood, expectations of future self-harm behaviors, as well as psychological factors implicated within the suicidal pathway, such as sense of entrapment (ie, lack of escape from unbearable thoughts, feelings, or situations) [26,27]. It also incorporated an exploratory, qualitative component in which participants provided open-ended feedback on the stories to assess their acceptability as potential interventions. The other study in this mixed methods project conducted focus groups with a subset of the participant sample used in this study (after they took part in the online experiment) to further explore this topic. The results from the focus group, published before this paper was written, demonstrated that although it is a complex relationship, individuals with recent self-harm experience could positively engage with lived experiences stories, for example, if they reflected a wide range of personal journeys and avoided the use of stigmatizing language [23].

## Methods

### Overview

This paper presents the quantitative and qualitative results of a self-administered online experiment [23] which explored how people with self-harm experience and interpret lived experience accounts as a means of support. The protocol [28] was preregistered ahead of data collection [29]. For results of the follow-up focus groups, see Winstone et al [23] for further discussion.

### Ethical Considerations

Ethical approval was granted by the University of Bristol School of Psychological Science Research Ethics Committee (reference number 10504). Before beginning the online experiment, participants were presented with the participant information sheet, which included researcher contact details. Participants were invited to contact the researcher with any questions regarding the study, before providing informed consent. Participants were able to navigate away from the survey while awaiting researcher response and resume participation once ready. Participants were then asked to confirm (via tick boxes) that they met the eligibility criteria and that they were providing informed consent to take part in the online study. Participants who completed the online survey were offered the option to enter a prize draw to win one of four £50 (US $67.50) Love2shop vouchers. All aspects of the General Data Protection Regulation and the Data Protection Act 2018 were adhered to. Anonymous study data have been backed up on a secured University of Bristol network drive and transferred to a designated University of Bristol Research Data Storage Facility for long-term archiving. The study data will be kept for a minimum of 15 years. The data sheet has been made open using the University of Bristol Research Data Repository.

### Recruitment

The study was advertised by the research team and mental health charities on their web pages, social media accounts, and in newsletters sent out to individuals who signed up to hear about regular research/charity activity. The study was also advertised on self-harm/mental health-specific forums, on Reddit, RecoverYourLife, and the National Self-Harm Network website. The advertisements contained a link to the experiment, which was programmed and hosted online using Gorilla Experiment Builder [30]. Participants needed access to a device and an internet connection, but could access the task from any device that allowed them to access any web browser. After confirming eligibility and providing consent, and completing a self-administered baseline questionnaire, participants were randomly assigned (using Gorilla’s balanced randomization feature) to one of the following three story conditions: (1) self-help strategies, (2) informal/formal help-seeking, (3) no help-seeking.

We conducted an a priori power analysis using G*Power (version 3.1.9.7; Heinrich-Heine-Universität Düsseldorf) [31], which determined that a sample of 246 participants would provide adequate (80%) power for our main analysis (1-way ANOVA) to detect a small to medium effect size (*f*=0.2) for our primary outcome (differences in help-seeking intentions) among the 3 story groups.

The study was conducted according to the revised Declaration of Helsinki (2013) and the 1996 International Council for Harmonisation Guidelines for Good Clinical Practice E6(R2). Further details about anticipated risks and our strategy to mitigate any risks can be found under “Ethical Considerations and Informed Consent” and “Safety” in the study protocol [28]. To be eligible for the study, participants had to be aged 16 years or older, fluent in English, currently resident in the United Kingdom, have engaged in self-harm within the past year, and be able to provide informed consent. Before beginning the study, participants had to provide informed written consent. Safeguarding resources were signposted to participants at the start and at the end of the study. Participants were asked to download a copy (or make note of) the safeguarding resources before beginning the study to ensure that all participants had access to the resources regardless of how far they progressed in the study. Participants were informed that it would take approximately 30 minutes to complete the survey. It was mandatory to answer all questions during the experiment, but participants were reminded that they could stop taking part at any time. Participants were also informed that the study was anonymous and that all data from participants who completed the study would be made public at the end of the study. After taking part, participants were debriefed (and again presented with safeguarding resources) and were offered the opportunity to enter a prize draw to win one of four £50 (US $67.50) Love2shop vouchers.

### Materials

A total of 3 template stories were used in this study (Multimedia Appendix 1). The same 3 templates were presented to all participants, but a portion of text was modified (removed and/or inserted) to reflect the different experimental conditions: self-help strategies, informal/formal help-seeking, and no help-seeking. The template stories were chosen from an initial shortlist of 10 real-life stories curated by BM. These were selected from a variety of online sources, including the websites of third-sector organizations, health care organizations, web-based forums, and personal blogs. This approach was used to ensure that they were not solely representative of the more polished stories that often appear on formal help sites. For ethical reasons, stories were excluded if they encouraged self-harm, described self-harm methods in detail, or did not have a recovery-oriented/hopeful outlook. Each of the 10 stories was independently ranked (in order of preference) and discussed by BM and 3 of the study authors (JF, IP-V, and LB). The 3 lived experience stories were then chosen following the ranking and discussion to be used as the template stories. The only key difference across the experimental conditions was whether text concerning self-help strategies, informal/formal help-seeking, or no help-seeking (but a positive/hopeful message) was inserted into the stories. These inserts were drawn from the original 10 stories selected by BM.

### Measures

#### Participant Characteristics (Assessed at Baseline)

Participants reported their age in years, their gender, sexual orientation, and ethnicity. They also answered questions on self-harm thoughts (past year frequency), passive and active suicidal thoughts (in lifetime and recency), nonsuicidal self-harm (in lifetime, recency, and past year frequency), and suicide attempts (in lifetime and recency). To measure help-seeking history, participants completed a modified version of the Actual Help-Seeking Questionnaire (AHSQ) [32,33]. For further details, please refer to the protocol [28].

#### Outcome Measures (Assessed Post Randomization)

##### Primary Outcome: Help-Seeking Intentions

To measure help-seeking intentions, participants completed a modified version of the General Help Seeking Questionnaire (GHSQ) [34]. A total GHSQ score was computed by summing responses across all help-source items, excluding the “I would not seek help from anyone” item, in line with prior research [35,36]

##### Secondary Outcomes: Affect, Entrapment, and Future Self-Harm

Participants completed the Positive and Negative Affect Schedule (PANAS) [37]. They completed the 4-item Entrapment Scale Short Form (E-SF) [38]. They also answered 2 questions about future self-harm, rated on a 5-point scale. First, how likely they would be to hurt themselves on purpose in the future after reading the stories, and second, how likely it was that they could see a time in the future without self-harm.

#### Open-Ended Feedback on the Lived Experience Stories

Participants were asked to provide open-ended feedback on the lived experience stories presented to them during the study. Specifically, they were asked, “Was there anything you found helpful/unhelpful about the stories? If so, please explain.”

### Attention Check

To ensure that only data from participants who were paying attention during the study were included in the analysis, one of the items among the outcome measures asked participants to select 1 on a scale from 0 to 4. Participants who selected a number other than 1 had their data removed prior to the analysis [39].

### Procedure

Participants completed the questionnaires on demographics, current/past self-harm thoughts and behaviors, and help-seeking history. They were then randomized, using Gorilla’s balanced randomization feature, to 1 of the 3 conditions (ie, self-help, informal/formal help-seeking, or no help-seeking). Participants were presented with and asked to read the 3 lived experience stories, with amended text based on the condition that they were assigned to. Participants were not aware of the condition they had been assigned to, nor were they informed that there were different variations of the stories. The stories were presented in the same fixed order for all participants. They then completed the outcome measures in the following order: (1) free text opinions of the lived experience stories, (2) mood, (3) entrapment, (4) thoughts of self-harm/suicide, and (5) help-seeking intentions.

### Analysis Plan

#### Data Screening

To maintain data quality, each participant’s data were screened against the following criteria prior to analysis: (1) each participant must have completed the entire study and (2) must have correctly answered the eligibility questions and the attention check question within the experiment. The data were assessed for normality using skewness and kurtosis statistics, and were assessed to ensure that they met the assumptions for ANOVA testing.

#### Data Analysis

Descriptive statistics were used to summarize the participant characteristics and outcome measures in each group. An ANOVA was used to test if the type of help-seeking mentioned in the lived experience story influenced the primary outcome of interest (ie, total score on the GHSQ). A series of ANOVAs was used to test if the type of help-seeking mentioned in the lived experience story influenced the secondary outcome measures (ie, total scores on PANAS, E-SF, and the 2 items assessing future self-harm). Where the type of help-seeking mentioned in the stories impacted any of the outcomes, post hoc pairwise comparisons were conducted using Tukey’s Honestly Significant Difference (HSD) test to control for multiple comparisons.

This study was powered to detect a main effect of the type of lived experience story; therefore, any interactions were exploratory. Where any of the analyses indicated a main effect of type of lived experience story on the outcome measures, then “story type × self-harm history” analyses of covariance (ANCOVAs) were used to assess if the influence of type of lived experience story on the outcome measures differed by self-harm history. The self-harm history variable was defined as the recency of self-harm behavior in the past year. In cases where a participant had a history of both nonsuicidal self-harm and attempting suicide, the recency score (indicating “either in the past week” or “more than a week ago, but in the past year”) for whichever behavior was more recent was used. Where any interactions existed, they were explored in post hoc analyses using 2-tailed *t* tests. As prior research demonstrates that gender [40,41], age [40], and prior help-seeking experience [42] impact future help-seeking, all analyses were run both unadjusted and adjusted for age, gender, and a continuous variable of help-seeking history (ie, total score on the AHSQ).

In the protocol, we had specified that exploratory analyses would be guided by the qualitative results. Findings from focus groups suggested that lived experience stories can have a positive impact on readers, but that this effect may be moderated by age; for example, older participants struggled emotionally when reading about someone else’s recovery that occurred by a certain age [23]. Therefore, we have included a tertiary analysis, exploring interactions between story type and age on any primary and secondary outcomes where main effects were found.

Participant opinions on the lived experience stories were analyzed using content analysis. After reviewing the data, specifically noting that many responses were brief and lacked richness, it was decided that instead of conducting thematic analysis (as originally specified in the protocol), it would be more appropriate to conduct content analysis. Established guidelines for conducting a conventional concept analysis were followed [43]. No preconceived categories were imposed; instead, key concepts were allowed to emerge inductively from the data. The data were imported into NVivo (version 14; QSR International) [44] for coding. Initial open coding was conducted independently by JF and JS (see Acknowledgments section), who identified salient thoughts and concepts within participants’ responses. JF then refined the coding scheme by examining conceptual relationships and consolidating codes where responses reflected similar underlying ideas. LW subsequently coded 10% of the responses, adding additional codes when deemed appropriate. Intercoder reliability was then assessed and calculated to be around 99%. JF reviewed any amendments, and any disagreements were discussed and resolved. Finally, JF refined and renamed codes to ensure that each label accurately captured the underlying concept.

## Results

The data that form the basis of the results presented here are available from the University of Bristol’s Research Data Repository [45].

### Participant Characteristics (Assessed at Baseline)

#### Demographics

Data collection ceased when there were complete datasets from 246 participants. One participant was identified as completing the study twice (based on IP address) and was removed from the dataset. Of the remaining 244 participants, data cleaning procedures identified 2 participants who failed the attention check and 6 who did not meet the eligibility criterion of having hurt themselves on purpose in the past year. Therefore, data from 238 (97.5% of our target sample size) participants were included in the analysis. Participants were primarily female (188/238, 78.9%), White (207/238, 86.9%), and were aged between 16 and 59 (mean 23.2, SD 9.4) years. Table S1 in Multimedia Appendix 2 provides further details on participant demographics across the experimental conditions, as well as a breakdown by experimental condition. The experiment ran for 28 weeks (from late March to early October 2022). In total, 381 individuals visited the link for the experiment, 379 provided informed consent, 361 passed the screening, and 340 were randomized.

#### Self-Harm Thoughts and Behaviors

**Figure 1:**
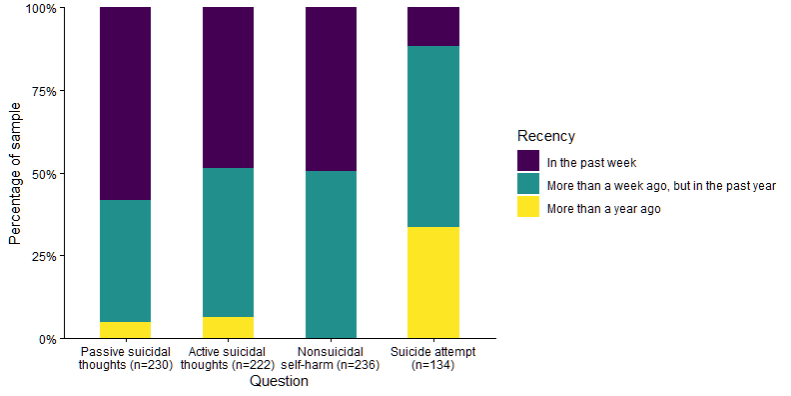
Recency of suicidal thoughts and self-harm behavior.

**Figure 2:**
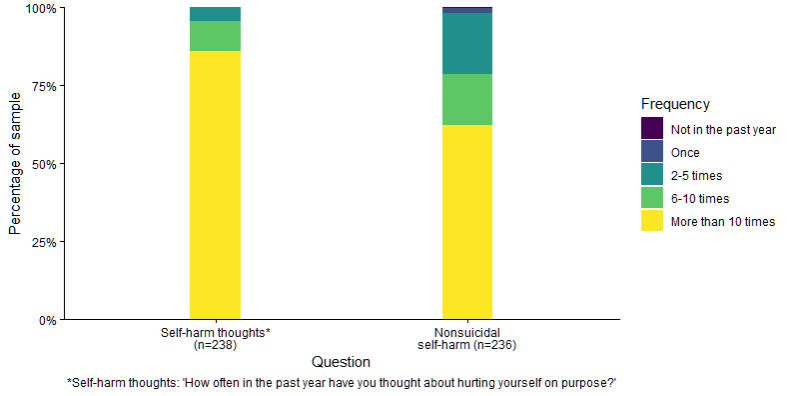
Frequency of self-harm thoughts and behavior (in the past year).

Out of 238 participants, 236 (99.2%) had engaged in nonsuicidal self-harm, 230 (96.6%) had thoughts that life was not worth living (ie, passive suicidal thoughts), and 222 (93.3%) had experienced suicidal ideations (ie, active suicidal thoughts) during their lifetime. Just over half the sample (n=134, 56.3%) had made a suicide attempt. Figures 1 and 2 display information about the recency and past-year frequency of self-harm thoughts and behaviors in the sample.

In terms of the type of self-harm methods used, 105 (44.1%) out of 238 participants reported swallowing pills or something poisonous, 109 (45.8%) reported burning themselves, and 226 (94.9%) reported cutting themselves. Using another method not listed was reported by 109 (45.8%) participants. These were screened by JF and BM and then categorized (for full details, please refer to Table S2 in Multimedia Appendix 2). Behaviors not considered to be self-harm (eg, tattooing, eating behaviors, or indirect self-harm such as unsafe sex) were removed; however, all participants reporting these behaviors also reported at least one other self-harm method, and so no participants were removed from the analysis. In terms of the number of self-harm methods used, 100 (42%) out of 238 participants reported having used 2 self-harm methods during their lifetime, with 45 (18.9%), 62 (26.1%), and 31 (13%) reporting 1, 3, and 4 or more methods, respectively.

#### Help-Seeking History

When reflecting on help-seeking over their lifetime, not seeking help from anyone was reported by 39 (16.4%) out of 238 participants. While some of these participants responded affirmatively to other items on the scale, 30 (12.6%) of those participants did not report any form of help-seeking. For a breakdown of the prevalence of accessing each source of informal and formal help, please refer to Table S3 in Multimedia Appendix 2.

### Outcome Measures (Assessed Post Randomization)

#### Overview

Descriptive statistics are reported for the 5 outcome measures by experimental condition in Table 1.

**Table 1:** Descriptive statistics for outcomes.

			Self-help	Informal/formal help	No help
**Primary outcomes, mean (SD; range)**					
	Help-seeking intentions (higher scores indicate greater intention to seek help),		34.3 (12.2; 13-73)	34.3 (12.3; 13-76)	38.6 (12.4; 13-73)
**Secondary outcomes, mean (SD; range)**					
	Positive affect		18.4 (6.0; 10-36)	18.0 (6.5; 10-43)	18.8 (6.0; 10-35)
	Negative affect		24.8 (10.4; 10-48)	28.5 (10.6; 11-49)	28.7 (9.5; 10-49)
	Entrapment		15.1 (4.9; 4-20)	15.0 (4.4; 4-20)	15.6 (4.1; 5-20)
	**Future self-harm, median (IQR)**				
		“After reading these stories, how likely is it that you will hurt yourself on purpose in the future?” (0=much less likely; 4=much more likely)	2 (2-2)	2 (2-2)	2 (2-3)
		“Can you see a time in the future without self-harm?” (0=not at all likely; 4=very likely)	2 (1-3)	1 (0-3)	1 (1-3)

#### Help-Seeking Intentions

**Figure 3:**
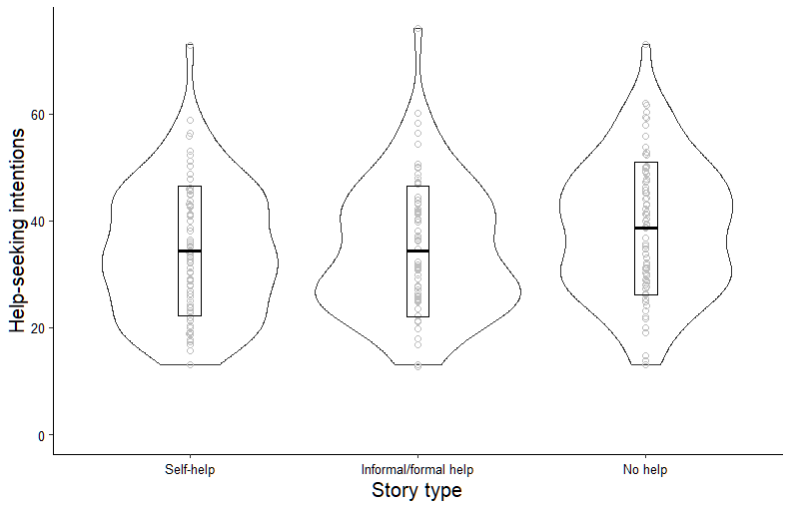
Impact of story type on help-seeking intentions.

Due to the skewed nature of the data, the variable for help-seeking intentions was transformed using a square-root transformation. There was moderate evidence for an effect of story type on help-seeking intentions (*F*_2, 235_=3.3; *P*=.04; η^2^=0.03). Adjusting for age, gender, and help-seeking history provided stronger evidence for an effect of story type on help-seeking intentions (*F*_2, 230_=4.2; *P*=.02; η^2^=0.25; the full results of the model using transformed and untransformed data can be found in Tables S1 and S2 in Multimedia Appendix 3). None of the pairwise comparisons (Tukey’s HSD on the unadjusted means) reached the conventional significance threshold. However, 2 comparisons approached significance, suggesting a trend toward group differences that were consistent with the overall effect. Participants in the “no help” condition reported marginally higher levels of help-seeking intentions after reading the stories compared to participants in the “self-help” condition (mean difference=–0.36, 95% CI –0.75 to –0.03; *P*=.08) and the “informal/formal help” condition (mean difference=–.36, 95% CI –0.74 to –0.02; *P*=.07). The impact of story type on help-seeking intentions is illustrated in Figure 3 (using untransformed data to aid interpretability).

An ANCOVA was conducted to determine whether self-harm history (recency of self-harm) interacted with story type to influence help-seeking intentions. Evidence for this interaction was not found (*F*_2, 232_=0.28; *P*=.76). An ANCOVA was conducted to explore if age interacted with story type to influence help-seeking intentions. Evidence for this interaction was also not found (*F*_2, 232_=1.25; *P*=.29). However, the former only achieved 15% power, and the latter 41% power.

#### Affect, Entrapment, Future Self-Harm

Due to the skewed nature of the data, attempts to normalize the distribution of all secondary outcomes, using square-root and cube transformations, were made. Despite these efforts, visual inspection of residuals and formal tests of normality indicated that the residuals remained nonnormal. Therefore, the following analyses were conducted using untransformed data.

We found no evidence for an effect of story type on positive affect (*F*_2, 235_=0.32; *P*=.73; η^2^=0.002) or entrapment (*F*_2, 235_=0.33; *P*=.72; η^2^=0.003). There was also no evidence for an effect of story type on future self-harm (likelihood of self-harm after reading stories: *F*_2, 235_=2.40; *P*=.09; η^2^=0.02; likelihood of a future without self-harm: *F*_2, 235_=0.94; *P*=.39; η^2^=0.008). The pattern of results was unchanged following adjustment for age, gender, and help-seeking history (the full results of the model can be found in Tables S3 and S5-S7 in Multimedia Appendix 3).

**Figure 4:**
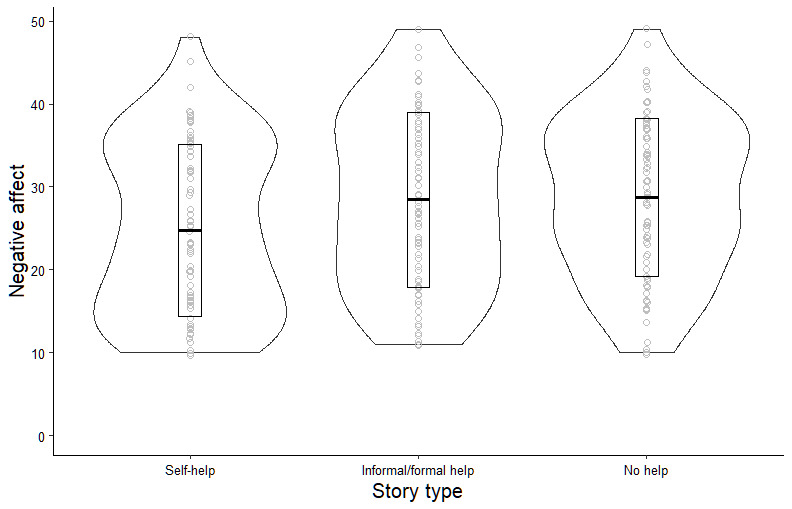
Impact of story type on negative affect.

There was evidence for an effect of story type on negative affect (*F*_2, 235_=3.83; *P*=.02; η^2^=0.03). Adjusting for age, gender, and help-seeking history increased the strength of association between story type and negative affect (*F*_2, 230_=4.02; *P*=.02; η^2^=0.10; the full results of the model can be found in Table S4 in Multimedia Appendix 3). Pairwise comparisons (Tukey’s HSD on the unadjusted means) demonstrated that participants in the “self-help” condition reported lower levels of negative affect after reading the stories compared to participants in the “no help” condition (mean difference=–3.97, 95% CI –7.72 to –0.22; *P*=.04) and the “informal/formal help” condition (mean difference=–3.70, 95% CI –7.55 to 0.14; *P*=.06). Figure 4 presents the impact of story type on negative affect.

An ANCOVA was conducted to explore whether self-harm history interacted with story type to influence negative affect. Although there was strong evidence for an effect of self-harm history on negative affect (*F*_1, 232_=28.94; *P*<.001), evidence for an interaction with story type was not found (*F*_2, 232_=0.94; *P=*.39). An ANCOVA was also conducted to explore if age interacted with story type to influence negative affect. Although there was strong evidence for an effect of age on negative affect (*F*_1, 232_=16.85; *P*<.001), evidence for an interaction was not found (*F*_2, 232_=1.06; *P*=.35). However, the former only achieved 22% power, and the latter 26% power.

#### Open-Ended Feedback on the Lived Experience Stories

Approximately 87.8% (209/238) of the sample provided written feedback on the stories. Limitations and advantages of the lived experience stories, categorized by the type of story, are reported in Tables S1 and S2 in Multimedia Appendix 4. Below, we have reported noticeable differences in the themes across conditions.

#### Differences in Perceived Limitations of the Stories Across Conditions

### The Recovery Messaging Was Problematic

Within the comments, messages of hope, positivity, and recovery were described as annoying, frustrating, and overwhelming. Some participants expressed dislike toward references to counting the number of days without self-harm. Other comments referred to the difficulty in looking to the future, as that felt dismissive and more challenging than taking it day by day. A participant explained,

I don’t care if I’m fine by the time I’m 21. I’ve ruined my teenage years with this, and I can’t get that back. Yeah, I hope for a happier future, but when people say that everything is fine because they recovered (...) they’re ignoring how horrific the emotions were when they were struggling. Recovery doesn’t magically erase the past.

Participant in “informal/formal help” condition

Another participant expressed frustration that the narrators in the stories seemed to be working toward recovery due to feeling stigmatized and pressured to conform, rather than of their own accord. The “no help” condition stands out in that more participants appeared to struggle with the inclusion of recovery messaging compared to the other story conditions. This may be because the recovery message appeared without a clear indication of how recovery occurred, as comments reflected the following sentiments,

Sometimes words can get lost, eg, “things get better.” The term has been used so many times that I have almost become desensitized to it and what it means.

Participant in “no help” condition

Doesn’t say how to get better.

Participant in “no help” condition

### The Stories Were Unrelatable

While some participants in all conditions commented on the stories being unrelatable, this was less pronounced in the “no help” condition. This is plausibly a result of the stories in this condition providing less concrete detail about the narrator’s experience, and thus, there being less to be able to relate to. Stories in the “self-help” condition were criticized more than stories in the other conditions for neglecting to acknowledge that “one size does not fit all.” This was mostly reflected in the criticism that the stories were too specific, usually because of a focus on younger ages. However, some participants in the “self-help” condition agreed with the sentiment that “the people in the stories seemed to have a better idea of why they started self-harming and why they continued to do it, which I don’t have.”

The most prevalent reasons why the stories were unrelatable referred to the stories having unrealistic portrayals of self-harm, and instilling feelings of competition and thus feelings of isolation in readers. They also commented that they felt pressure to have “stronger urges” and “scars” as the narrators had. Participants in all conditions found the stories unrealistic to a similar degree; however, more references about the stories seeming sanitized or inauthentic were made by participants in the “self-help” condition. Participants in the “self-help” and “informal/formal help” conditions commented on the stories, making them feel isolated much more than those in the “no help” condition. Several participants emphasized that there is a very competitive nature to self-harm at both extremes:

It can sometimes make you feel as if you are not bad enough to deserve help.

Participant in “self-help” condition

All people who seemed to be able to stop with help. It made me feel bad for still struggling.

Participant in “informal/formal help” condition

### The Specific Support Mentioned in the Stories (or Lack Thereof) Was Problematic

The stories in the “self-help” condition were criticized for not mentioning professional or medical support. All conditions were criticized for offering nonpractical advice. This was more predominant in the “self-help” condition, followed by the “informal/formal help” condition, and much less in the “no help” condition. The type of self-help methods mentioned were mainly criticized for being methods that readers had already tried and found unsuccessful in aiding recovery; there were also a couple of comments criticizing the focus on short-term coping mechanisms, instead of long-term recovery.

After trying the methods they said helped them, I eventually slipped back to self harming. I don’t know what else to do.

Participant in “self-help” condition

They didn’t give much detail on what actually helped them other than immediate coping mechanisms.

Participant in “self-help” condition

The type of informal/formal help methods mentioned were mainly criticized, as participants felt the stories made accessing those kinds of support sound much easier than it is. For example, a participant stated,

Sometimes it may not be as easy to access support services, whilst barriers to access are likely to vary, it felt like the people in the stories were able to somewhat easily access support - the way it should be I guess.

Participant in “informal/formal help” condition

Another stated,

I’m a first-generation immigrant, mental health was never something to talk about in my household because of cultural reasons. If I had gone to my parents, I know I wouldn’t get the same sympathy just frustration.

Participant in “informal/formal help” condition

This made readers who did not have supportive friends/family or who had struggled to access formal support feel isolated and distressed,

I guess it makes me sad to hear about people having their boyfriend, friends and family around to help them when I feel like I have no one.

Participant in “informal/formal help” condition

#### Differences in Perceived Advantages of the Stories Across Conditions

While the “informal/formal help” condition did not stand out in the number of criticisms received, participants in this condition did seem to have fewer positive things to say about the stories. This was apparent in relation to comments about the stories having a realistic but hopeful outlook of recovery and comments about the authenticity of the stories.

### The Stories Included a Realistic But Hopeful Outlook on Recovery

Participants in all conditions appreciated hearing the stories from those who recovered, and particularly the emphasis on recovery being a nonlinear process that takes time. Only the “self-help” stories were praised for highlighting that the path to recovery is not a one-size-fits-all approach, and that readers will find different coping mechanisms and recovery strategies helpful. As mentioned earlier, participants in the “self-help” condition were also more likely to criticize the stories for failing to do exactly this. Only the stories in the “self-help” and “no help” conditions were praised for the discussion of relapses, despite these themes appearing in all conditions.

### The Stories Were Relatable

The number of positive comments about the relatability of the stories was similar across conditions. However, there were some differences among the conditions when looking at the subthemes of relatability. Although positive comments about the authenticity of the stories were made about all of the stories, this was more prevalent in the “self-help” and “no help” conditions. All of the stories were commended for accurately portraying the emotions experienced in self-harm, but the stories in the “self-help” and “no help” conditions were further commended for relaying “lived experience” and seeming “honest and truthful.” One participant explained that,

It can be hard to accept advice from people that don’t fully understand or appreciate just how dark the thoughts can be and how strong these urges and negative thoughts can take over your mind.

Participant in “no help” condition

We note that there are inconsistencies between the unrelatable and relatable themes, which we will elaborate on in the discussion.

### The Stories Were Supportive

Unsurprisingly, participants in the “no help” condition had fewer positive remarks about the theme of support compared to participants in the conditions that focused on help-seeking. However, this difference was not as substantial, as some participants in this condition felt that hearing others’ stories reminded them that they were not alone. It is also worth noting that this feeling of “camaraderie” was reported a similar number of times across conditions. Other aspects that fell under the theme of support seen in the other conditions were: participants in the “self-help” condition appreciated hearing what specific methods worked for others, as it gave them hope, generally, but also ideas about new coping mechanisms to try. Participants in the “informal/formal help” condition appreciated the emphasis on the importance of accessing support for recovery. The stories highlighted to them how helpful it was to have both informal and formal help at the start, but also throughout recovery. The stories could help to instill hope and confidence,

It does get better once you find the right support. There are people who do care about you, you just don’t know it.

Participant in “informal/formal help” condition

While some participants who had negative or no help-seeking experiences in the past found it difficult to read about others having success, some participants found it reassuring to hear about other people successfully accessing support, as there is concern that accessibility can be restricted.

## Discussion

### Main Findings

In this study of individuals with recent self-harm experience, we were unable to find clear evidence of an association between the type of content of lived experience stories and help-seeking intentions, entrapment, or positive affect. However, we did find evidence that reading stories about self-help strategies led to a lower prevalence of negative emotions, compared to reading stories about seeking help from others, or stories that made no mention of help-seeking.

One potential explanation for why we did not see an increase in help-seeking intentions after reading a story that emphasizes help-seeking might be due to the relatability of the stories. The open-ended feedback suggested that the stories that mentioned help-seeking were seen as less relatable than the stories that did not mention help-seeking. This demonstrates that it is not only the characteristics of the narrator (eg, age and gender) that the reader might not relate to. They might also struggle to relate to the actions and consequences of help-seeking experienced by the narrator. First, about 12.6% (30/238) of participants reported no history of help-seeking. Others who recounted negative experiences when they sought help (eg, ineffective self-help strategies, negative reactions from family and friends, and inaccessible formal support) reported feeling distressed (ie, feeling like a failure, alone, and hopeless) when they read about others’ positive experiences. Further, only the stories that mentioned help-seeking seemed to evoke a feeling that the reader’s situation was less serious than the narrator’s. As perceived need is an important component of help-seeking decision-making [46], it is feasible that the stories that mentioned help-seeking set a benchmark for when help-seeking is needed. In our focus group study, we similarly found that being able to identify with the narrator of a lived experience story—made more challenging by including specific details of age or circumstances—was “an important factor in encouraging help-seeking through the recognition of need in others and thus oneself” [23].

Findings need to be interpreted in light of some limitations. First, the sample was self-selecting, so it is possible it may not be representative of the wider population; further, there was a considerable degree of attrition, and the reasons for this are unknown. Second, we did not measure help-seeking intentions before and after the exposure, so we cannot determine if there was an overall increase across all participants. Third, the study was powered to detect a main effect of the type of lived experience story, and tests of interactions were exploratory and underpowered. These post hoc findings therefore need to be interpreted with caution, and larger-scale research is needed to understand the role of moderators, such as self-harm history and age. Fourth, as we only measured help-seeking intentions, we do not know if these intentions would manifest into help-seeking behavior. Therefore, future research would benefit from recruiting a larger sample, capturing reasons for deciding not to participate or withdrawing from the study, and including a follow-up assessment to measure whether intentions translated into behavior.

It is worth noting that some of the themes that emerged from the open-ended feedback questions contradicted one another. For example, the self-help stories seemed to arouse thoughts about self-harm and recovery being very individualistic experiences, but they were both praised for doing this well and critiqued for doing it poorly. Similarly, the same aspect of a story could be a positive that made the story more relatable for one participant, and a negative addition that made the story less relatable for another. While some attempts were made to reconcile contradictions like this when interpreting the data, we were conservative in our approach. First, because we felt that the contradictions were the result of a broad focus. Feedback was collected from a large number of individuals, and that feedback could be directed toward various aspects of the stories. Second, the contradictions in themselves may be meaningful, as they indicate how individualistic the experience of self-harm and recovery can be, and emphasize that a delicate balance needs to be found in these stories if hoping to apply them broadly.

Overall, there was broad consistency between the themes that emerged from the open-ended feedback collected in this study and those that emerged during the focus group discussions with 5.5% (13/238) of the original sample [23]. Both studies highlighted the importance of lived experience stories to be relatable, but that a careful balance had to be struck between specificity and ambiguity. Both studies also highlighted the importance of lived experience stories to feel authentic (avoiding both dramatization and sanitization) and that authenticity is a key factor in how relatable a story is to the reader. Previous research on mental health recovery narratives also emphasizes the importance of authenticity to readers [47,48]. A systematic review on peer support for self-harm found that shared experience was an important component in making the support they received feel genuine [49]. While the template stories were written by those with lived experience, it was up to the research team’s discretion about how to modify them for the purpose of this study, and this might have impacted the relatability or other aspects of the stories. Therefore, we suggest using a co-design approach in future work.

In line with the findings of Winstone et al [23], this study also highlighted the importance of lived experience stories to offer practical advice and to portray the journey to recovery realistically. This finding might be specific to self-harm recovery, as another study, which more broadly focused on participants with any mental health concern and excluded narratives that described harmful behavior (eg, self-harm), found participants reported a preference for narratives about upward trajectories toward recovery over narratives that focused on only snapshots of the journey or nonlinear journeys [47].

Results from the focus groups suggest that reading the stories had a generally positive impact on mood [23]. We did not measure baseline mood in this study, and we cannot assume that if we did that the results would support the qualitative findings, as the focus group sample may have been subject to sampling bias, as participants who felt the stories negatively impacted their mood might have been less likely to enroll in the follow-up qualitative study. Nonetheless, this study did demonstrate that participants who read stories about self-help strategies reported lower levels of negative emotions, compared to the other two groups. The open-ended feedback suggested that reading about self-help strategies that were successful for others left some readers feeling hopeful that recovery might be possible for them too. It also gave them ideas of new strategies to try, inspiring them to act toward achieving their own recovery. It is possible that these positives may have increased their self-efficacy [50] to recover, thereby increasing their mood and decreasing feelings of needing to rely on others for help. While this interpretation is speculative, further investigation is warranted since an unintended negative consequence may be a decreased likelihood of seeking help from others.

The results from this study suggest that readers might react more negatively to themes of recovery in the absence of practical advice on how to recover. The results from this study also add the caveat that it would be beneficial to mention various help-seeking strategies within the same story. This might prevent the reader from feeling demoralized (and avoid seeking help) after reading about someone else successfully obtaining help via a strategy that was unsuccessful for the reader or is a strategy that they are unwilling to try.

Our findings, consistent with the findings of Winstone et al [23], demonstrate that lived experience stories can help readers to “feel validated and help them to better understand their own feelings and experiences” and manage “feelings of isolation, knowing that others are experiencing similar feelings, and engaging in self-harm as a coping mechanism” [23]. However, the results from both studies also highlight that reading about others’ experiences can sometimes foster a sense of competition and thereby increase feelings of isolation. Previous research has also highlighted the competitive element of self-harm [19,42,49] and found that individuals sometimes purposefully seek out material that instills feelings of competition when experiencing self-harm urges [42].

One way to prevent lived experience stories from triggering a sense of competition in readers is by avoiding words such as “clean” or other language that might cause readers to feel stigmatized or under pressure to recover. Focus group participants in the follow-up study further explained that stigma can arise when using these terms, because they can unfairly attach moralistic judgments to a medical issue [23]. In addition, it might be helpful to explicitly refer, in lived experience stories, to the competitive nature of self-harm. For example, narrators can emphasize that they are relaying only one out of many stories (that not everyone’s self-harm will have the same causes, that not everyone will experience it in the same way, and that not everyone will recover in the same way), but that everyone’s experience is valid. Winstone et al [23] further concluded that avoiding numeric references within a story, such as the number of days without self-harm, might help to reduce competition.

The lived experience stories under study were carefully selected to be recovery-oriented and exclude details that might be triggering for someone who self-harms (eg, detailed descriptions of self-harm methods). While this was an important consideration to mitigate risk in this study, it does mean that the results may not generalize to lived experience stories that include more triggering content that one might find on unregulated forums. This will need to be kept in mind if using the results of this study to inform practice.

Further, despite attempts to exclude triggering content from the stories, the results demonstrated that some participants (across all conditions) reported emotional distress as a result of reading the stories. Some participants were negatively impacted by the mention of the specific self-harm method used (ie, cutting). While lived experience stories on forums often include descriptive detail about methods and injuries [51], those hosted on more formal websites (eg, run by charities and health care organizations) usually exclude these kinds of details [22]. However, some participants were negatively impacted by other details that might be found in stories hosted on formal websites, such as hearing about others struggling with self-harm, being reminded of how they harm themselves, or how one’s self-harm might impact their loved ones. It was also reported that reading about others still struggling with self-harm made readers feel hopeless that their situation would ever meaningfully improve. Therefore, future research, using a co-design approach, is needed to identify elements that make a story harmful.

### Conclusions

Lived experience stories of self-harm are commonly found on the internet, including on formal help sites, blog posts, and forums. The results of this study provide guidance for those posting or moderating such content. The findings taken together illustrate that the impact of lived experience stories on individuals with a recent history of self-harm is complicated. While the inclusion of help-seeking in a story can help readers to feel validated, less isolated, less helpless, and more hopeful that recovery is possible for them, there is a fine line to tread. The results indicate that the experience of self-harm and the pathway to recovery are very personal, so too many personal details about either experience can make the stories feel unrelatable. However, there are certain shared experiences of self-harm that can be included to ensure a lived experience story feels authentic. Therefore, the results suggest that stories should aim to foster both a sense of individuality as well as community. For example, stories should focus less on the characteristics of narrators, but more on their emotions and experience of self-harm, to which readers can more easily relate. Stories should also propose multiple strategies for achieving recovery, and emphasize that recovery will take time and that there may be setbacks along the way. Future experimental work is needed to confirm whether these adaptations to lived experience stories about help-seeking can motivate individuals to seek help for their self-harm. Future experimental work should also incorporate suggestions on how to limit bias and improve the methodology used in this study. Lived experience stories of self-harm that find the right balance of many of these nuances have the potential to encourage those struggling with self-harm urges to engage in self-help practices or reach out to others for support.

## Appendix

Multimedia Appendix 1 Story templates.

Multimedia Appendix 2 Participant characteristics.

Multimedia Appendix 3 Outcome measures.

Multimedia Appendix 4 Open-ended feedback.

## Abbreviations

AHSQ: Actual Help-Seeking Questionnaire
ANCOVA: analysis of covariance
E-SF: 4-item Entrapment Scale Short Form
GHSQ: General Help Seeking Questionnaire
HSD: Honestly Significant Difference
PANAS: Positive and Negative Affect Schedule
