# Supplementary material for: Investigating the Impact of Lived Experience Stories on Self-Harm, Mood, and Help-Seeking Intentions: Web-Based Between-Participants Experimental Study in Individuals With Recent Self-Harm
Source: JMIR Hum Factors. 2026 Mar 4;13:e71280. doi: 10.2196/71280 (PMC13000381; doi:10.2196/71280)
Supplement: Multimedia Appendix 1 [file humanfactors_v13i1e71280_app1.docx]

## Multimedia Appendix: Story templates

Text in black is the story template which is consistent across study conditions. Text in blue was inserted to reflect the three different themes 1) self-help, 2) informal/formal help and 3) no help.

### Story template S1

Source: [Emma's story | Samaritans](https://www.samaritans.org/wales/how-we-can-help/if-youre-having-difficult-time/if-you-want-self-harm/emmas-story/).

The self-help and informal/formal help inserts are from the original story and the no help insert is from a blog post (link not provided).

#### 1) Self-help condition

I started self-harming when I was 15. I was bullied really badly, and I was so lonely. I didn’t understand why I was getting picked on. I thought it had to be me that’s the problem, and that’s when I started to self-harm and think about suicide.

I carried so much anger with me all of the time. When I lost all my friends, that anger had to go somewhere. It seemed like the easiest thing was to inflict pain on myself. I saw it as a punishment. I thought I was such a bad person.

Nowadays, to manage my emotions, I read a lot: I always have self-care books on my phone. If I feel anxious, it’s about physically bringing myself back into the room. I smell something, taste something and touch something. I do breathing exercises, and once I feel like I’m ok I put on something to do with self-help, on iTunes, Amazon or YouTube: something that is gentle but is inspiring.

I read a lot about mindfulness, accepting where you are in life. I’ve found that when you accept the here and now, life will become a lot easier. That’s incredibly powerful for me.

When I was self-harming, if someone had said to me when you are 21, you are going to be happy and healthy, I wouldn’t have believed them.

But here I am.

#### 2) Informal/formal help condition

I started self-harming when I was 15. I was bullied really badly, and I was so lonely. I didn’t understand why I was getting picked on. I thought it had to be me that’s the problem, and that’s when I started to self-harm and think about suicide.

I carried so much anger with me all of the time. When I lost all my friends, that anger had to go somewhere. It seemed like the easiest thing was to inflict pain on myself. I saw it as a punishment. I thought I was such a bad person.

I’ve had talking therapy and thought a lot about why I self-harmed. It was so helpful for my recovery. Now I can recognise what I’m triggered by. When it first started, it was the pain of being so on my own and so angry. As I got older, it all revolved around relationships: when my ex-boyfriend finished with me, or when I fell out with my mum.

My mum spoke for me because I was too fragile. Everyone else kept trying to brush it off and telling me to cheer up. I was lucky to have someone to say you are really, really struggling and I want to help you.

When I was self-harming, if someone had said to me when you are 21, you are going to be happy and healthy, I wouldn’t have believed them.

But here I am.

#### 3) No-help condition

I started self-harming when I was 15. I was bullied really badly, and I was so lonely. I didn’t understand why I was getting picked on. I thought it had to be me that’s the problem, and that’s when I started to self-harm and think about suicide.

I carried so much anger with me all of the time. When I lost all my friends, that anger had to go somewhere. It seemed like the easiest thing was to inflict pain on myself. I saw it as a punishment. I thought I was such a bad person.

Regardless of whether you have had incidents of self-harm or self-destructive thoughts, whatever your age, or for those of you who have felt the siren call of ‘ending it all’, those of you who know what true hopelessness feels like. I hope you find the help you need, **the hope**we all need. **Sometimes just waiting things out — this one night, this one moment, this one week, this one month — that can do the trick. Things ease up. I swear.**

When I was self-harming, if someone had said to me when you are 21, you are going to be happy and healthy, I wouldn’t have believed them.

But here I am.

### Story template S2

##### Source: [Interview with Maddie Bruce: recovering from self-harm - The Mix](https://www.themix.org.uk/mental-health/self-harm/interview-with-maddie-bruce-whats-it-like-to-recover-from-self-harm-35672.html).

##### The informal/formal help insert and a portion of the no help insert are from the original story. The other part of the no help insert is from a post on recover your life [Recoveryourlife.com - Home](https://www.recoveryourlife.com/). The self-help insert is from a website containing personal stories of self-harm recovery: [Self harm: our stories, and how we recovered | By Sian Abigail Bradley | Medium](https://sianabradley1.medium.com/self-harm-our-stories-and-how-we-recovered-31c160d8dae8) (those wishing to access this page now need to sign-up to medium.com)

#### 1) Self-help condition

I don’t think I realised that what I was doing was self-harming until I was about 16 (I started at 14). I had figured it out as a way to hurt/punish myself and I kept it secret for a long time.

There were lots of relapses but because I was putting the effort in and working on myself, I was using healthy coping techniques instead and things seemed to improve overall.

The first thing I do is go on a walk and just get moving. There’s a lot of apps. Calm Harm is a popular one that was recommended to me. I find that something as simple as Solitaire and Sudoku also helps, as they engage your brain. I also listen to the sleep stories on Calm. I do find that calms me down.

Another thing that really helps is drawing the same thing over and over again. Just to keep my hands moving as much as anything. And on that note, I do always like to have something with me to fiddle with. Because I can get these thoughts and feelings 24/7, anytime, anyplace. So this helps me manage the intense stress and uncomfortable feeling that comes with a barrage of intrusive thoughts and urges to hurt yourself.

Relapses can be disheartening because you can feel like you’ve made so much progress by being say 40 days clean and then you break it and it can feel like you’re back to square one. But instead of pressuring myself to be 41 days clean the next time I would just focus on staying clean for however long I could.

Finally, it is possible. I didn’t think I‘d be able to do it in a million years but it does get easier over time. Be patient, stay strong and you will get there, I promise.

#### 2) Informal/formal help condition

I don’t think I realised that what I was doing was self-harming until I was about 16 (I started at 14). I had figured it out as a way to hurt/punish myself and I kept it secret for a long time.

My mum walked in on me doing it, she was hysterical but then I opened up to her and said it had been something I’d been doing since I was 14 and that it was a way of me punishing myself or something I did to get a release. After finding out, my parents then took me to the doctor to get help. The doctor then referred me to mental health services.

There were lots of relapses but because I was putting the effort in and working on myself, I was using healthy coping techniques instead and things seemed to improve overall. Relapses can be disheartening because you can feel like you’ve made so much progress by being say 40 days clean and then you break it and it can feel like you’re back to square one. But instead of pressuring myself to be 41 days clean the next time I would just focus on staying clean for however long I could.

My friends were super supportive. Throughout my recovery I’ve met lots of people who also struggle with the same issues so having them to speak to was great. But even my friends who had never struggled gave me their time if I was feeling urges and wanted to talk through how I was feeling. My family, and especially my mum, were amazing. They kept the house as safe as possible for me. My mum was just a non-judgemental comforting influence at that time. I think she realised there was nothing she could do to stop it and even though it made her sad she was just supportive and that’s all I needed at the time.

Finally, it is possible. I didn’t think I‘d be able to do it in a million years but it does get easier over time. Be patient, stay strong and you will get there, I promise.

#### 3) No-help condition

I don’t think I realised that what I was doing was self-harming until I was about 16 (I started at 14). I had figured it out as a way to hurt/punish myself and I kept it secret for a long time.

There were lots of relapses but because I was putting the effort in and working on myself, I was using healthy coping techniques instead and things seemed to improve overall. Relapses can be disheartening because you can feel like you’ve made so much progress by being say 40 days clean and then you break it and it can feel like you’re back to square one. But instead of pressuring myself to be 41 days clean the next time I would just focus on staying clean for however long I could.

Be easy on yourself because recovery is not linear. It’s forwards, backwards, up, down and round and round but when you commit to getting yourself to a better headspace and remember that self-harm is not the answer to all your problems, things will get better. The setbacks shouldn’t matter as much, it’s how you pick yourself back up again.

It's totally okay to miss it and grieve for the loss of something that was a big part of who you were at one point in time. That's totally fine. And again it doesn't mean it has to be a thing you keep doing. You can feel those things without having to act on the urges.

Finally, it is possible. I didn’t think I‘d be able to do it in a million years but it does get easier over time. Be patient, stay strong and you will get there, I promise.

### Story Template S3

##### Source: Personal Blog (link not provided)

##### The informal/formal help insert is from the original blog post. The self-help insert is from a website containing personal stories of self-harm recovery: [Self harm: our stories, and how we recovered | By Sian Abigail Bradley | Medium](https://sianabradley1.medium.com/self-harm-our-stories-and-how-we-recovered-31c160d8dae8). The no-help insert is from two separate stories combined on [Recoveryourlife.com - Home](https://www.recoveryourlife.com/).

#### 1) Self-help condition

Around 2010/2011, I was getting very stressed about life in general. My parents were pushing me to find a job, among other things. Over the course of a few months, it got to the point where I was starting to feel like I wasn’t living up to their expectations, and that I was worthless. At that point, I started feeling numb, and I started cutting. I had been feeling emotionally numb at the time, and that I was losing control of my life. Cutting gave me a sense of control over something even as I felt like my life was falling apart around me.

My mom was the first one to really notice how my behaviour changed, and along with it my personality. I wasn’t really interested in the things I had been interested in, I was eating and sleeping more, and was pretty much moping around the house. Every time she would ask me something, I would answer with “I don’t know” or “probably not” about 90% of the time.

It took me a couple of months before I realised that I needed help. Something that I found to really help me, which may sound cliche, was journaling. Using words, scribbles, doodles as an outlet when things got to the point of feeling too much. I would also paint on my body as a temporary distraction.

I used to rip apart thick cardboard boxes whenever I felt desperate to self-harm. I was advised to snap a bobble around my wrist instead or to hold ice within my hands and clench my fists, but I found these awfully unhelpful and not at all something I engaged in

It’s not linear, but I have always been a creative person and creating something or taking my urges out in a creative way was for me, the only thing that released and allowed me to actually get to a point where I stopped self-harming.

I have not thought about cutting for the last 7 years. I have no plans of getting rid of the scars from then, as I see them as a reminder of how I had hit a low point in my life and survived.

#### 2) Informal/formal help condition

Around 2010/2011, I was getting very stressed about life in general. My parents were pushing me to find a job, among other things. Over the course of a few months, it got to the point where I was starting to feel like I wasn’t living up to their expectations, and that I was worthless. At that point, I started feeling numb, and I started cutting. I had been feeling emotionally numb at the time, and that I was losing control of my life. Cutting gave me a sense of control over something even as I felt like my life was falling apart around me.

My mom was the first one to really notice how my behaviour changed, and along with it my personality. I wasn’t really interested in the things I had been interested in, I was eating and sleeping more, and was pretty much moping around the house. Every time she would ask me something, I would answer with “I don’t know” or “probably not” about 90% of the time.

It took me a couple of months before I realised that I needed help. My mom and I went to see a few therapists, but the first two I saw I didn’t really connect with. The third therapist I pretty much immediately clicked with. During the initial meeting I was told I had clinical depression. I was put on medication, and I continued to see my therapist throughout the whole ordeal, which helped.

Initially not much seemed to change. I still had thoughts of cutting when things got really stressful. After a few months, though, I started noticing the changes. Then, I started dating my current boyfriend. Early on in the relationship, I did tell him that I used to cut and that I had been diagnosed with depression. Whenever I had a bad episode or got really upset, I would either text or call him and he would talk to me to help me think of something else other than cutting. That definitely helped.

Now I am off the medication, and have not thought about cutting for the last 7 years. I have no plans of getting rid of the scars from then, as I see them as a reminder of how I had hit a low point in my life and survived.

#### 3) No-help condition

Around 2010/2011, I was getting very stressed about life in general. My parents were pushing me to find a job, among other things. Over the course of a few months, it got to the point where I was starting to feel like I wasn’t living up to their expectations, and that I was worthless. At that point, I started feeling numb, and I started cutting. I had been feeling emotionally numb at the time, and that I was losing control of my life. Cutting gave me a sense of control over something even as I felt like my life was falling apart around me.

My mom was the first one to really notice how my behaviour changed, and along with it my personality. I wasn’t really interested in the things I had been interested in, I was eating and sleeping more, and was pretty much moping around the house. Every time she would ask me something, I would answer with “I don’t know” or “probably not” about 90% of the time.

I have not thought about cutting for the last 7 years. I have no plans of getting rid of the scars from then, as I see them as a reminder of how I had hit a low point in my life and survived.

It isn't fast, it isn't simple, and it isn't easy, but recovering your life is worth it. YOU are worth it. Day by day, week by week, month by month, year by year, keep fighting. Please keep fighting.

I have found that over time the thoughts lessen. I am of the belief that they will never entirely go away but what changes is the severity and the impact that they have on your life. I know that I get thoughts about self-injury but they're that -just thoughts. I hope that you find inner peace with yourself.
