# Supplementary material for: Investigating the Impact of Lived Experience Stories on Self-Harm, Mood, and Help-Seeking Intentions: Web-Based Between-Participants Experimental Study in Individuals With Recent Self-Harm
Source: JMIR Hum Factors. 2026 Mar 4;13:e71280. doi: 10.2196/71280 (PMC13000381; doi:10.2196/71280)
Supplement: Multimedia Appendix 2 [file humanfactors_v13i1e71280_app2.docx]

## Multimedia Appendix: Participant characteristics

**Table S1.** Participant demographics.

|  | *All*  *(n=238)* | | *Self-help (n=83)* | *Informal/*  *formal help (n=75)* | *No help (n=80)* |
| --- | --- | --- | --- | --- | --- |
|  |  | |  |  |  |
| ***Age M(SD)*** | 23.2(9.4) | | 23.4(9.6) | 21.6(8.2) | 23.8(10.2) |
|  |  | |  |  |  |
| ***Gender*** | **n** | **%** | **%** | **%** | **%** |
| Female | 188 | 79 | 79 | 73 | 84 |
| Male | 25 | 11 | 9 | 16 | 7 |
| Prefer to self-describe^a^ | 20 | 8 | 10 | 9 | 6 |
| Prefer not to say | 5 | 2 | 3 | 1 | 2 |
|  |  |  |  |  |  |
| ***Ethnicity*** | **n** | **%** | **%** | **%** | **%** |
| Arab | 2 | 1 | 1 | 1 |  |
| Asian/Asian British | 4 | 2 | 1 | 4 |  |
| Black/African/Caribbean/Black British | 6 | 2 | 4 |  | 4 |
| White | 207 | 87 | 81 | 87 | 93 |
| Mixed/multiple ethnic groups | 17 | 7 | 12 | 7 | 2 |
| Other ethnic group^b^ | 1 | <1 |  | 1 |  |
| Prefer not to say | 1 | <1 |  |  | 1 |
|  |  |  |  |  |  |
| ***Sexual orientation*** | **n** | **%** | **%** | **%** | **%** |
| Asexual or Aromantic  (attracted to no genders) | 11 | 5 | 5 | 5 | 4 |
|  |  |  |  |  |  |
| Bisexual or pansexual  (attracted to all genders) | 88 | 37 | 36 | 39 | 36 |
|  |  |  |  |  |  |
| Gay or lesbian  (attracted to people of the same gender) | 23 | 10 | 12 | 8 | 8 |
|  |  |  |  |  |  |
| Straight  (attracted to people of the opposite gender) | 97 | 41 | 35 | 40 | 47 |
|  |  |  |  |  |  |
| Prefer to self-describe^c^ | 10 | 4 | 6 | 3 | 4 |
| Prefer not to say | 9 | 4 | 5 | 5 | 1 |
| ^a^Agender, Genderfluid, Genderqueer, Non-binary, Non-binary AFAB, She/They, Trans female to male, Transmasculine  ^b^Portuguese  ^c^Not straight, Not sure, Omnisexual, Pansexual, Queer and/or lesbian, Sapphic, Unlabelled | | | | | |

**Table S2.** Self-harm methods reported.

|  | |
| --- | --- |
| 1. Attempting to get hit by traffic or jump from a high place | Standing on/jumping from bridge or high place |
|  | Standing in traffic |
| 2. Strangulation | Hanging, ligature strangulation and manual strangulation |
| 3. Blood loss | Blood letting |
| 4. Causing pain/injury to skin, skin appendages, blood vessels, bones, muscles, joints, nerves | Hitting, slapping, punching, scratching, pinching, biting, bruising, burning (using boiling water, friction, or stinging nettles) oneself with their hands or an object |
|  | Dropping heavy objects on the body or using heavy objects to crush parts of the body/break bones |
|  | Punching objects or banging head/limbs against objects or scraping them against rough surfaces |
|  | Inserting sharp/foreign objects into body |
|  | Dislocating joints on purpose |
|  | Electric shocks |
|  | Nail removal |
|  | Putting chemicals on wounds |
| 5.Swallowing harmful items | Swallowing non-food items |
|  | Overdosing |

**Table S3.** Help-seeking history.

|  | **%** | **n** |  |
| --- | --- | --- | --- |
| ***Informal sources*** |  |  |  |
| a friend | 59 | 141 |  |
|  |  |  |  |
| school/university staff (e.g., guidance counsellor, teacher, academic advisor, professor) | 28 | 67 |  |
|  |  |  |  |
| a partner | 26 | 62 |  |
|  |  |  |  |
| a parent | 18 | 43 |  |
|  |  |  |  |
| another relative or family member | 8 | 19 |  |
|  |  |  |  |
| ***Formal sources*** |  |  |  |
| a mental health professional (e.g., counsellor, psychologist or psychiatrist) | 54 | 135 |  |
|  |  |  |  |
| a phone helpline or chat service (e.g., Samaritans) | 45 | 108 |  |
|  |  |  |  |
| a doctor or GP | 43 | 102 |  |
|  |  |  |  |
| an online support/forum | 37 | 87 |  |
|  |  |  |  |
| Accident and Emergency | 22 | 52 |  |
|  |  |  |  |
| another professional (e.g., social worker, nurse) | 13 | 32 |  |
|  |  |  |  |
| a community group (e.g. a religious group or a support group) | 11 | 27 |  |
|  |  |  |  |
| ***Other sources*** |  |  |  |
| a source of support other not listed | 1 | 2 | Chaplain/priest, self-harm charity |
