# Supplementary material for: Investigating the Impact of Lived Experience Stories on Self-Harm, Mood, and Help-Seeking Intentions: Web-Based Between-Participants Experimental Study in Individuals With Recent Self-Harm
Source: JMIR Hum Factors. 2026 Mar 4;13:e71280. doi: 10.2196/71280 (PMC13000381; doi:10.2196/71280)
Supplement: Multimedia Appendix 3 [file humanfactors_v13i1e71280_app3.docx]

## Multimedia Appendix: Outcome measures

### ANCOVAs

**Table S1.** Impact of story type on future help-seeking (untransformed data) adjusting for age, gender, and help-seeking history.

| **Predictor** | **df** | **Sum of squares** | **Mean Square** | **F** | ***P*** |
| --- | --- | --- | --- | --- | --- |
| **Story type** | 2 | 988 | 494 | 4.20 | .02 |
| **Age** | 1 | 84 | 84 | 0.72 | .40 |
| **Gender** | 3 | 185 | 62 | 0.52 | .67 |
| **Help-seeking history** | 1 | 8068 | 8068 | 68.60 | < 0.001 |
| **Error** | 230 | 27050 | 118 |  |  |

**Table S2.** Impact of story type on future help-seeking (transformed data) adjusting for age, gender, and help-seeking history.

| **Predictor** | **df** | **Sum of squares** | **Mean Square** | **F** | ***P*** |
| --- | --- | --- | --- | --- | --- |
| **Story type** | 2 | 7.10 | 3.55 | 4.22 | .02 |
| **Age** | 1 | 0.42 | 0.42 | 0.50 | .48 |
| **Gender** | 3 | 1.09 | 0.36 | 0.43 | .73 |
| **Help-seeking history** | 1 | 57.35 | 57.35 | 68.21 | < 0.001 |
| **Error** | 230 | 193.40 | 0.84 |  |  |

**Table S3.** Impact of story type on positive affect adjusting for age, gender, and help-seeking history.

| **Predictor** | **df** | **Sum of squares** | **Mean Square** | **F** | ***P*** |
| --- | --- | --- | --- | --- | --- |
| **Story type** | 2 | 24 | 12.02 | 0.32 | 0.72 |
| **Age** | 1 | 65 | 65.34 | 1.76 | 0.19 |
| **Gender** | 3 | 49 | 16.34 | 0.44 | 0.72 |
| **Help-seeking history** | 1 | 279 | 278.62 | 7.52 | 0.007 |
| **Error** | 230 | 8523 | 37.06 |  |  |

**Table S4.** Impact of story type on negative affect adjusting for age, gender, and help-seeking history.

| **Predictor** | **df** | **Sum of squares** | **Mean Square** | **F** | ***P*** |
| --- | --- | --- | --- | --- | --- |
| **Story type** | 2 | 788 | 394.0 | 3.42 | .02 |
| **Age** | 1 | 1626 | 1625.7 | 9.01 | < 0.001 |
| **Gender** | 3 | 62 | 120.7 | 0.46 | .89 |
| **Help-seeking history** | 1 | 1 | 0.7 | 0.37 | .93 |
| **Error** | 230 | 22521 | 97.9 |  |  |

**Table S5.** Impact of story type on entrapment adjusting for age, gender, and help-seeking history.

| **Predictor** | **df** | **Sum of squares** | **Mean Square** | **F** | ***P*** |
| --- | --- | --- | --- | --- | --- |
| **Story type** | 2 | 14 | 6.76 | 0.34 | .71 |
| **Age** | 1 | 178 | 178.18 | 9.01 | .003 |
| **Gender** | 3 | 27 | 9.00 | 0.46 | .71 |
| **Help-seeking history** | 1 | 3 | 7.36 | 0.38 | .54 |
| **Error** | 230 | 4546 | 19.77 |  |  |

**Table S6.** Impact of story on the likelihood of future self-harm in the short-term adjusting for age, gender, and help-seeking history.

| **Predictor** | **df** | **Sum of squares** | **Mean Square** | **F** | ***P*** |
| --- | --- | --- | --- | --- | --- |
| **Story type** | 2 | 2.86 | 1.43 | 2.42 | .09 |
| **Age** | 1 | 3.12 | 3.12 | 5.48 | .02 |
| **Gender** | 3 | 0.42 | 0.14 | 0.24 | .87 |
| **Help-seeking history** | 1 | 0.55 | 0.55 | 0.93 | .34 |
| **Error** | 230 | 135.67 | 0.59 |  |  |

**Table S7.** Impact of story type on the likelihood of a future without self-harm in the long-term adjusting for age, gender, and help-seeking history.

| **Predictor** | **df** | **Sum of squares** | **Mean Square** | **F** | ***P*** |
| --- | --- | --- | --- | --- | --- |
| **Story type** | 2 | 3.4 | 1.70 | 0.94 | .40 |
| **Age** | 1 | 7.5 | 7.50 | 4.12 | .05 |
| **Gender** | 3 | 2.6 | 0.88 | 0.48 | .70 |
| **Help-seeking history** | 1 | 0.2 | 0.20 | 0.11 | .74 |
| **Error** | 230 | 418.9 | 1.82 |  |  |

## 
