# Supplementary material for: Investigating the Impact of Lived Experience Stories on Self-Harm, Mood, and Help-Seeking Intentions: Web-Based Between-Participants Experimental Study in Individuals With Recent Self-Harm
Source: JMIR Hum Factors. 2026 Mar 4;13:e71280. doi: 10.2196/71280 (PMC13000381; doi:10.2196/71280)
Supplement: Multimedia Appendix 4 [file humanfactors_v13i1e71280_app4.docx]

## Multimedia Appendix: Open-ended feedback on the lived experience stories

**Table S1.** Limitations of the lived experience stories.

| For the overarching themes, we have reported the number of references made for that theme in each condition. If a participant’s comments were coded under more than one subtheme of an overarching theme, they were represented more than once in the total.  For subthemes, we have not reported the number of references made for that theme, but only denoted whether or not it applied to each condition. Subthemes are listed in order of prevalence. | | Theme mentioned by participants in: | | |
| --- | --- | --- | --- | --- |
|  |  | Self-help condition | Informal/formal help condition | No help condition |
| **The stories were unrelatable** | | **32** | **27** | **21** |
| The stories had an unrealistic portrayal of self-harm | Readers felt the stories were sanitised/inauthentic. It was pointed out that lived experience stories are only over about those who survive | x | x |  |
|  | The stories made recovery sound easier than it is. The stories did not mention that it can be non-linear process and a lifelong struggle | x | x | x |
| The stories instilled feelings of competition and thus feelings of isolation | Hearing that narrators have had success using coping strategies, responding to help, and/or recovering when the reader is still struggling made readers feel guilty, shameful, like a failure, hopeless, and under pressure to recover  Hearing that narrators have support systems when the reader has no one to help them made readers feel alone and upset  Hearing about different causes of self-harm, different levels of severity, or the mention of scars, made readers feel like their situation is not legitimate or severe enough to receive help | x | x | x |
| The details in the stories were too specific | Readers found it difficult to relate to narrators who are at a different stage of recovery to them (e.g., a narrator who wants to get better or is already better) | x |  | x |
|  | Readers found it difficult to relate to stories that focused on younger years | x | x | x |
|  | Readers found it difficult to relate to narrators who had clear causes for self-harm, had urges to self-harm, or had scars | x |  |  |
| The details in the stories were too vague | Readers found it difficult to relate to the narrators because their age was not specified |  | x |  |
|  | Readers found it difficult to relate to the narrators because the severity of their self-harm was not specified | x |  |  |
| **The stories caused emotional distress** | | **9** | **6** | **8** |
| The specification of method (i.e., cutting) was triggering | | x | x | x |
| Readers were upset by reading about harm to oneself and others | Readers were upset by reading about others battling self-harm | x | x |  |
|  | Readers were upset by reading about how one’s self-harm impacts their loved ones |  | x |  |
|  | Readers were upset by being reminded of how they harm themselves |  |  | x |
| Readers struggled to believe that things will get meaningfully better, particularly when they read about those who are still struggling | | x |  | x |
| **The specific support mentioned in the stories (or lack thereof) was problematic** | | **11** | **6** | **3** |
| The stories did not provide novel strategies for getting help; only mentioned methods that readers have tried and found did not work for them or have not tried because they knew they would not work for them | | x | x |  |
| The stories made accessing support from friends/family and formal support services sound easier than it is | |  | x |  |
| The stories did not specify how the narrators received help | |  |  | x |
| The stories did not mention professional help | | x |  |  |
| **The recovery messaging was problematic** | | **2** | **3** | **11** |
| Criticisms of recovery messaging in general | Readers find messages of hope, recovery, day counting to be annoying, frustrating, or overwhelming | x |  | x |
|  | Readers are desensitised to recovery messaging |  |  | x |
|  | The stories ignored the fact that recovery does not erase the past |  | x |  |
| Criticisms of recovery messaging in these stories specifically | The stories focused on long-term recovery instead of tackling it day by day |  | x |  |
|  | The narrators seemed to be recovering solely for others’ benefit |  | x |  |
|  | The stories lacked details about how the narrators recovered | x |  |  |
| **The presentation style was problematic** | | **4** | **5** | **3** |
| The tone of the stories was… | Dismissive | x | x |  |
|  | Patronising |  | x |  |
| The wrong language was used in the stories (e.g., "clean", "relapses", "recovery", “promise") | |  | x | x |
| The length of the stories was too short | | x | x |  |
| Words (or text) were the wrong medium (for example, it can be difficult to express the feelings of self-harm using words; solid text can be disengaging) | |  | x | x |
| **The narrators did not adequately describe why they started to self-harm** | | **6** | **3** | **0** |
| The stories did not acknowledge self-harm as a coping mechanism for underlying mental health issues and therefore their focus seemed superficial (i.e., how to manage the symptoms but not address the cause) | | x | x |  |
| The stories oversimplified the thoughts and feelings experienced before and during self-harm, making them sound easy to address/overcome | | x | x |  |
| **The stories were not useful** | | **1** | **2** | **2** |
| **These types of stories are cliché** | | **2** | **2** | **1** |
| **The stories could have negative impact dependent on the reader’s mood** | | **1** | **0** | **1** |

**Table S2.** Advantages of the lived experience stories.

| For the overarching themes, we have reported the number of references made for that theme in each condition. If a participant’s comments were coded under more than one subtheme of an overarching theme, they were represented more than once in the total.  For subthemes, we have not reported the number of references made for that theme, but only denoted whether or not it applied to each condition. Subthemes are listed in order of prevalence. | | Theme mentioned by participants in: | | |
| --- | --- | --- | --- | --- |
|  |  | Self-help condition | Informal/formal help condition | No help condition |
| **The stories includes realistic but hopeful outlook of recovery** | | **44** | **22** | **40** |
| The stories were hopeful and confidence-boosting | | x | x | x |
| Readers found it helpful to hear from those that recovered, and those that recovered even when they thought they never would | | x | x | x |
| Readers found it help to read that even if the urges never completely go away, it can get easier | |  |  | x |
| The stories portrayed barriers to recovery | The stories highlighted that recovery is non-linear | x | x | x |
|  | The stories highlighted that not every help strategy will work for everyone | x |  |  |
| **The stories were supportive** | | **34** | **31** | **19** |
| The stories offered peer support | Knowing that others go through what they do made readers feel less alone; readers found it helpful to read about what has helped others | x | x | x |
| The stories gave examples of support and highlighted why and how to get help | The stories highlighted the importance of support from others |  | x |  |
|  | The stories made readers aware of ways to access support | x | x |  |
|  | It was reassuring for readers to hear that some people have found ways to cope without relying on others | x |  |  |
|  | The stories gave them new ideas for ways to manage their symptoms and aid their recovery | x | x |  |
| **The stories were relatable** | | **19** | **17** | **22** |
| Readers related to the stories generally, the causes of self-harm, or the emotions of the narrators | | x | x | x |
| Readers related to the coping methods used by the narrators | | x |  |  |
| Readers related to the consequences of self-harm, the pressure to recover, and experiencing negative reactions from others | |  | x |  |
| Readers related to the feeling of missing self-harm during recovery | |  |  | x |
| The stories were not too specific | The stories did not go into details like causes of self-harm |  |  | x |
|  | Having multiple stories meant readers could related to aspects from all three |  |  | x |
| The stories felt authentic | It felt obvious that the stories were written by those with lived experience; Emotions were accurately portrayed; The stories felt truthful | x | x | x |
|  | The stories were not overdramatised |  | x |  |
| **The presentation style was beneficial** | | **5** | **2** | **3** |
| The character traits of narrator were inspiring | Courageous |  |  | x |
|  | Optimistic | x |  |  |
| The tone was understanding, non-judgmental, and self-accepting, especially in relation to scars and recovery progress | | x | x | x |
| The use of simple sentences in the stories made the messages easy to take in | | x | x | x |
| **Storytelling is beneficial** | | **1** | **2** | **2** |
| It was helpful for readers to have someone else recount an experience similar to their own | |  | x | x |
| It was helpful for readers to have someone else put their experience into words as it can be difficult to do it themselves | | x |  |  |
| It was helpful for readers to read stories that are often not heard in real life | |  | x |  |
| **The narrators described why they started to self-harm (and acknowledged self-harm as a coping mechanism for underlying mental health issues)** | | **2** | **0** | **3** |
| **The narrators described when they knew they had to stop self-harming** | | **0** | **0** | **1** |
| **The narrators described their experiences without going into graphic details about self-harm** | | **0** | **1** | **0** |
